# Clinical care issues between safety and quality of care for the late preterm: "The Future of Nursing in Neonatology "

**DOI:** 10.1186/1824-7288-40-S2-A44

**Published:** 2014-10-09

**Authors:** Graziella Costamagna

**Affiliations:** 1 Director of Health Professions SC SITRO AO Mauriziano Turin and component technical group National Federation of Colleges IPASVI on 'Pediatric Nursing, Italy

## Neonatology and pediatric nursing in the world and in Europe

Professional standards of the American and Canadian companies stress the importance of a minimum number of "experts" nurses of neonatology or pediatrics in the reality where infants and children are assisted.

Also the document about PNAE of Education stresses how training programs for the general nursing in many countries don't give the necessary preparation to nurses in this area.

## The professional practice of neonatology and pediatric area in Italy

The Pediatric Nurse in Italy is the figure that the DM 70/1997 has identified as the one responsible of the nursing care to individuals in growth.

These are flanked by the generalist nurses with specialized annual training.

But also the general nurse according to DM 793/1994 can assist babies and children, as he's generally enabled to the nursing care of individuals of all ages.

Even the Midwife is enabled to care babies and also in delivery room.

## The reflections within the professional community about the future

The Federation IPASVI in the past 10 years has activated some strategies to support and enhance the innovation in this area of neonatology and pediatric care through the creation of working groups of pediatric nurses at the national level, the spred of best care practices in this area, the guidelines for the university training of master's degree in the pediatric area, the definition of competence's profiles of nurse in neonatal area differentiated by levels of complexity of care, a patnership with a SIN to the building of the certification's manual of the path birth of the group GINS/Agenas, allowing to the pediatric's area nurses to work on the network throughot the country.

The neonatology's and pediatric's scientific associations and of universities have allowed evidence of the specificity of this area and the development of best practices and evidence.

The reflection on the future of the neonatology's and pediatric's nurses in Italy is still open. Crucial points are the definition of uniform standards training and operating within the national territory, the skills certification, the paths of professional accreditation and professional skills and advanced responsibilities.

